# Urinary phytoestrogens and the risk of uterine leiomyomata in US women

**DOI:** 10.1186/s12905-023-02381-5

**Published:** 2023-05-13

**Authors:** Fang Yang, Youguo Chen

**Affiliations:** Department of Obstetrics and Gynecology, The First Affiliated Hospital of Soochow University, No.899 Pinghai Road, Suzhou, 215006 P. R. China

**Keywords:** Uterine leiomyomata, Urinary phytoestrogens, NHANES, Weighted quantile sum, Relationship

## Abstract

**Background:**

Uterine leiomyomata (UL) is a common gynecological disease in women. Studied on the relationship between single metabolites of urinary phytoestrogens and UL, especially for the combined effects of mixed metabolites on UL still are insufficient.

**Methods:**

In this cross-sectional study, we included 1,579 participants from the National Health and Nutrition Examination Survey. Urinary phytoestrogens were assessed by measuring urinary excretion of daidzein, genistein, equol, O-desmethylangolensin, enterodiol and enterolactone. The outcome was defined as UL. Weighted logistic regression was used to analyze the association between single metabolites of urinary phytoestrogens and UL. Notably, we adopted the weighted quantile sum (WQS) regression, Bayesian kernel machine regression (BKMR), and quantile g-computation (qgcomp) models, to investigate the combined effects of six mixed metabolites on UL.

**Results:**

The prevalence of UL was approximately 12.92%. After adjusting age, race/ethnicity, marital status, drinking status, body mass index, waist circumference, menopausal status, ovary removed status, use of female hormones, hormones/hormone modifiers, total energy, daidzein, genistein, O-desmethylangolensin, enterodiol, and enterolactone, the association of equol with UL was significant [Odds ratio (OR) = 1.92, 95% confidence interval (CI): 1.09–3.38]. In the WQS model, mixed metabolites of urinary phytoestrogen had a positive association with UL (OR = 1.68, 95%CI: 1.12–2.51), with the highest weighted chemical of equol. In the gpcomp model, equol had the largest positive weight, followed by genistein and enterodiol. In the BKMR model, equol and enterodiol have positive correlation on UL risk, while enterolactone has negative correlation.

**Conclusion:**

Our results implied a positive association between the mixed metabolites of urinary phytoestrogen and UL. This study provides evidence that urinary phytoestrogen-metabolite mixture was closely related to the risk of female UL.

**Supplementary Information:**

The online version contains supplementary material available at 10.1186/s12905-023-02381-5.

## Background

Uterine leiomyomata (UL) are the most common solid tumors in women [1, 2]. It is estimated that up to 80% of women will develop UL during their lifetime [1, 3], with 25–30% of them experiencing significant symptoms, including chronic pelvic pain, dysmenorrhea, abnormal vaginal discharge, and abnormal menstruation [3, 4]. UL continues to pose a serious disease burden for women.

Although the underlying pathology of UL is not particularly clear, it has been suggested to be an estrogen-dependent tumor [5]. Phytoestrogens are a group of plant compounds that are similar in chemical structure to mammalian estrogens, and they can be absorbed from food, circulate in the blood, and are excreted in the urine [6–8]. Previous studies have reported the effects of phytoestrogens on UL [9, 10]. For example, a case–control study included 328 eligible subjects from the Diagnostic Unit of the University Hospital of the West Indies, found that there was no association of urine daidzein, genistein, equol, enterolactone, total phytoestrogens and uterine fibroid (diagnosed by abdominal and/or vaginal ultrasonography) using binary logistic regression analysis [9]. A cross-sectional study contains 1,204 participants performed by Zhang Y, et al., implied that equol was significantly associated with the risk of UL after adjusting for age, race, pregnant status, ovary removed status, use of female hormones, body mass index (BMI), menopausal status and urinary creatinine levels [10]. There are still contradictions regarding the effects of phytoestrogens on uterine fibroids. Importantly, these studies on the association of phytoestrogens with UL have focused on the effects of single chemicals [9, 10]. Generally speaking, humans are often exposed to many chemicals simultaneously, and the cumulative effect of multiple chemicals is of concern [11]. Nevertheless, little is known about the mixed effects of multiple chemicals in phytoestrogens on UL.

Herein, this study aimed at investigating the relationship between single metabolites of urinary phytoestrogens and UL in US women, and the combined effects of mixed metabolites on UL risk.

## Methods

### Population selection

In this cross-sectional study, all data were drawn from the National Health and Nutrition Examination Survey (NHANES) database. The NHANES is a cross-sectional survey conducted by the National Center for Health Statistics (NCHS) of the Centers for Disease Control and Prevention using a multilayer probability sampling design, which aim to assess the health and nutritional status of adults and children in the United States [12]. The NHANES survey combines interviews and physical examinations [13]. The requirement of ethical approval and informed consent of the subjects for this was waived by the Institutional Review Board of The First Affiliated Hospital of Soochow University, because the data was accessed from NHANES (a publicly available database). All methods were carried out in accordance with relevant guidelines and regulations.

**Fig. 1:**
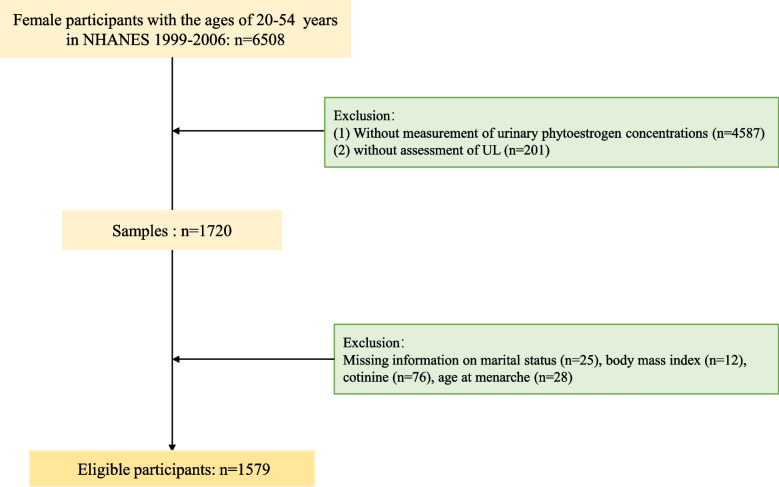
Flowchart of population selection. NHANES = National Health and Nutrition Examination Survey; UL = uterine leiomyomata

In this study, we used data from four cycles of the NHANES database (NHANES 1999–2000, 2001–2002, 2003–2004, 2005–2006). For participants in the NHANES database, only women aged 20–54 were asked diagnostic questions about UL (*n* = 6,508). Participants who met one of the following criteria were excluded: (1) Women without measurement of urinary phytoestrogen concentrations; (2) Women without assessment of UL; (3) Women with missing information of covariates related to UL. Ultimately, 1,579 participants were included in this study (Fig. 1).

### Assessment of urinary phytoestrogen

Urinary phytoestrogens were assessed by measuring urinary excretion of isoflavones (including daidzein, genistein, equol, and O-desmethylangolensin) and enterolignans (including enterodiol and enterolactone) [14]. The collection of urine specimens was carried out in the Mobile Examination Centers, and stored at -20 °C until analyzed [14]. The analyses of urinary excretion were accomplished by using the high-performance liquid chromatography (HPLC)-tandem mass spectrometric (MS) detection in the survey 1999–2004 and HPLC-atmospheric pressure photoionization- MS in the survey 2005–2006 [15]. For 1,579 participants of this study, 1 participant were below the lower limit of detection (LOD) for daidzein (0.40 ng/mL), 9 participants were below the lower LOD for genistein (0.20 ng/mL), 2 participants were below the lower LOD for equol (0.06 ng/mL), 29 participants were below the lower LOD for O-desmethylangolensin (0.20 ng/mL), 0 participants were below the lower LOD for enterodiol (0.04 ng/mL) and 0 participants were below the lower LOD for enterolactone (0.10 ng/mL) [16]. In the case of results below the LOD, the value of this variable is the LOD divided by the square root of two (https://wwwn.cdc.gov/Nchs/Nhanes/1999-2000/PHPYPA.htm#URXDAZ). The concentration of daidzein, genistein, equol, O-desmethylangolensin, enterodiol, and enterolactone in urinary phytoestrogens was corrected by creatinine in this study. Geometric mean and tertiles of each phytoestrogen metabolite (ug/g creatinine) were presented in Supplemental Table 1.

### Assessment of uterine leiomyomata

The outcome was considered as UL. Participants in the NHANES database were classified as patients with UL when they answered “Yes” to the question “Has a doctor or other health professional ever told you that you had uterine fibroids?”.

### Potential covariates

We extracted some characteristics of participants from the NHANES database, including age (years), race/ethnicity (non-Hispanic White/ non-Hispanic Black/ others), marital status (married/ never married/ others), education level [high school and below/ high school grad/ general educational development (GED) or equivalent/ some college or associate of arts (AA) degree/college graduate or above], poverty-to-income ratio (PIR, < 1.0/ ≥ 1.0), smoking status (yes/no), drinking status (yes/no), BMI (kg/m^2^), waist circumference (cm), cotinine (ng/mL), age at menarche (years), menopausal status (yes/no), ovary removed status (yes/no), hysterectomy (yes/no), use of female hormones (yes/no), hormones/hormone modifiers, pregnancy status (yes/no), number of gravidities, fiber (gm) and total energy (kcal). PIR was classified as in the NHANES database ≥ 1.0 (meaning household income was above the poverty line) and < 1.0 (meaning household income is at or below the poverty line). Smoking status and drinking status in the NHANES database was based on participants’ self‐report. BMI was calculated as weight (kg) divided by height squared (m^2^). Cotinine was assessed measured in serum using isotope dilution-high performance liquid chromatography/atmospheric pressure chemical ionization tandem mass spectrometry. Similarly, when the result is below the LOD, the value of cotinine is the LOD divided by the square root of two. Information on age at menarche, menopausal status, ovary removed status, use of female hormones, hormones/hormone modifiers, pregnancy status and number of gravidities was obtained from the reproductive health questionnaire. Use of female hormones was judged by self-report " Have you/Has SP ever used female hormones such as estrogen and progesterone?" and drug code 97–101 in the NHANES database. Hormones/hormone modifiers was defined according to drug codes [97–98, 97–103, 97–288, 97–295, 97–377, 97–411, 97–413, 97–414, 97–416, 97–417, 97–418, 97–420, 97–422, 97–423, 97–426, 97–495].

### Statistical analysis

Given the nature of the complex sampling of the NHANES database, we used a weighted analysis: weight variables for the urinary metabolites measurement (WTSB2YR and WTSPH2YR) and study design variables (SDMVPSU and SDMVSTRA). The measurement data were tested for normality using Kolmogorov–Smirnov, and normally distributed measurement data were described as mean (standard error) [Mean (SE)] and compared between two groups using independent samples t-test; non-normal data were described as median and quartiles [M (Q1, Q3)] and compared between groups using Mann–Whitney U rank sum test. Categorical data were described as number of cases and composition ratio N (%) and compared between groups using chi-square test and rank data using rank sum test. In the present study, we adopted chain equation multiple interpolation method based on random forest for some missing data of the variables. The miceforest package in python is used for interpolation processing (https://pypi.org/project/miceforest/). A sensitivity analysis was performed on the data before and after interpolation (Supplemental Table 2). SAS (version 9.4), Python (version 3.9) and R (version 4.0) software were used for statistical analyses. *P* < 0.05 was considered as statistically significant difference.

First, we performed weighted univariate logistic regression to screen covariates. Then, weighted logistic regression was used to analyze the association between single metabolites of urinary phytoestrogens and UL. Odds ratio (OR) and 95% confidence interval (CI) were calculated in the study. Last, we adopted three statistical models: weighted quantile sum (WQS) regression, Bayesian kernel machine regression (BKMR), and quantile g-computation (qgcomp) models, to investigate the effects of six mixed metabolites on UL.

### Weighted quantile sum (WQS) regression

WQS regression was used to investigate the effects of six mixed metabolites on UL and identify the predominant metabolite. The study sample was randomly divided into training dataset (30%, *n* = 474) and validation dataset (70%, *n* = 1,105). Exposure to each metabolite in the training dataset was first divided into tertiles. The tertiles were then added together to generate an overall tertiles score for each metabolite. An empirical weight for each metabolite in the mixture was estimated using the bootstrapping method [17]. The WQS score is a combination of six mixed metabolites, representing the whole-body burden of six urinary phytoestrogens [10]. The weight of each metabolite in the WQS score indicates the contribution of each metabolite to the overall result [18]. Metabolites with an estimated weight greater than 0.333 (1/3) were considered to be significant contributors to the WQS score. Using 10,000 bootstrap samples from the training dataset (30%), we calculated the weights for WQS scores. Using the validation dataset (70%), we assess the statistical significance of WQS scores [19]. In addition, WQS regression requires that all exposure-outcome associations be focused in the same direction. Therefore, we estimated the positive and negative effects of the six metabolites on UL separately. R package gWQS was adopted to perform the analysis.

### Quantile g-computation (qgcomp) model

gqcomp is a parameterized and generalized linear model based on application of g-computation, aimed to assess the effect of increasing all exposures in the mixture by one quatile simultaneously [20]. In this study, the gqcomp.noboot function was applied to estimate exposure effects, which divides six mixed metabolites into tertiles, assigns a positive or negative weight to each metabolite. If a metabolite has multiple effects in different directions, a positive or negative weight is interpreted as the proportion of exposure effects that have a negative (or positive) effect on UL, with a total weight of up to 2. The relationship of each metabolite endpoint and the mixed metabolites was assessed separately, and the finding models were used to estimate the scaled effect sizes, variable-specific coefficients, and overall model fit p-values. Metabolites with an estimated weight greater than 0.05 were considered to be significant contributors to the gqcomp scores. R package qgcomp was adopted to perform the analysis.

### Bayesian kernel machine regression (BKMR)

BKMR is a supervised approach, which could identify nonlinear and nonadditive associations of exposure-outcome [21]. In this study, the BKMR model with 10,000 iterations was adopted. Genistein, equol and enterodiol were divided into two groups according to their positive correlation with UL, while daidzein, O-desmethylangolensin, and enterolactone were divided into one group according to their negative correlation with UL. The combined effect was calculated by comparing mixed metabolites at or above the 60th percentile with the 50th percentile. Group posterior inclusion probability (GroupPIP) and Conditional posterior inclusion probability (CondPIP) represent the probability of each group and metabolite in each group included in the model, representing their contribution to the overall effect. R package bkmr was adopted to perform the analysis.

## Results

### Population characteristics

Table 1 presents the general characteristics of 1,579 eligible participants. The average age was 37.81 years. Approximately 69.00% of participants reported a history of drinking, and 32.14% of participants indicated that they were menopausal. In addition, all participants were divided into UL group (*n* = 204) and non-UL group (*n* = 1,375). Age, race/ethnicity, marital status, drinking status, BMI, waist circumference, menopausal status, ovary removed status, use of female hormones, hormones/hormone modifiers, number of gravidities and total energy were significantly different between UL group and non-UL group (*P* < 0.05).

**Table 1:** The general characteristics of included participants

**Variables**	**Total (*n* = 1579)**	**UL group (*n* = 204)**	**Non-UL group (*n* = 1375)**	***P***
Age, years, Mean (S.E)	37.81 (0.31)	44.54 (0.59)	36.68 (0.36)	< 0.001
Race/ethnicity, n (%)				< 0.001
Non-Hispanic White	745 (69.30)	90 (67.05)	655 (69.67)	
Non-Hispanic Black	314 (11.94)	76 (20.13)	238 (10.57)	
Other race^a^	520 (18.76)	38 (12.82)	482 (19.75)	
Marital status, n (%)				0.028
Married	913 (58.62)	124 (65.13)	789 (57.54)	
Never married	299 (17.72)	24 (9.98)	275 (19.01)	
Other^b^	367 (23.66)	56 (24.90)	311 (23.45)	
Education level, n (%)				0.783
High school and below	362 (13.79)	33 (11.91)	329 (14.10)	
High school Grad/ GED or Equivalent	334 (22.32)	42 (22.70)	292 (22.26)	
Some College or AA degree/College Graduate or above	883 (63.89)	129 (65.40)	754 (63.64)	
PIR, Mean (S.E)				0.113
< 1.0	314 (15.39)	23 (11.19)	291 (16.09)	
≥ 1.0	1265 (84.61)	181 (88.81)	1084 (83.91)	
Smoking status, n (%)				0.129
No	986 (56.77)	115 (50.34)	871 (57.85)	
Yes	593 (43.23)	89 (49.66)	504 (42.15)	
Drinking status, n (%)				0.045
No	611 (31.00)	68 (24.70)	543 (32.06)	
Yes	968 (69.00)	136 (75.30)	832 (67.94)	
BMI, kg/m^2^, Mean (S.E)	28.27 (0.28)	29.63 (0.58)	28.04 (0.29)	0.007
Waist circumference, cm, Mean (S.E)	92.67 (0.59)	95.54 (1.32)	92.20 (0.64)	0.020
Cotinine, ng/mL, Mean (S.E)	59.55 (4.30)	61.99 (10.57)	59.14 (4.71)	0.808
Age at menarche, years, Mean (S.E)	12.55 (0.06)	12.40 (0.14)	12.58 (0.06)	0.240
Menopausal status, n (%)				< 0.001
No	1030 (67.86)	86 (42.13)	944 (72.16)	
Yes	549 (32.14)	118 (57.87)	431 (27.84)	
Ovary removed status, n (%)				< 0.001
No	1455 (90.64)	132 (62.53)	1323 (95.33)	
Yes	124 (9.36)	72 (37.47)	52 (4.67)	
Hysterectomy, n (%)				< 0.001
No	1432 (88.85)	118 (55.22)	1314 (94.47)	
Yes	147 (11.15)	86 (44.78)	61 (5.53)	
Use of female hormones, n (%)				< 0.001
No	1270 (73.74)	128 (58.70)	1142 (76.26)	
Yes	309 (26.26)	76 (41.30)	233 (23.74)	
Use of other hormonal drugs, n (%)				0.035
No	1479 (91.46)	180 (86.89)	1299 (92.22)	
Yes	100 (8.54)	24 (13.11)	76 (7.78)	
Use of non-steroidal anti-inflammatory drugs, n (%)				0.844
No	1515 (95.06)	194 (94.74)	1321 (95.11)	
Yes	64 (4.94)	10 (5.26)	54 (4.89)	
Pregnancy status, n (%)				0.086
No	1297 (95.11)	193 (97.62)	1104 (94.69)	
Yes	282 (4.89)	11 (2.38)	271 (5.31)	
Number of gravidities, n (%)				0.002
1	216 (13.19)	21 (10.12)	195 (13.70)	
> 1	1125 (67.32)	165 (79.12)	960 (65.34)	
Unknown	238 (19.50)	18 (10.76)	220 (20.96)	
Daidzein, ug/g, Mean (S.E)	307.31 (38.08)	472.64 (148.81)	279.70 (36.63)	0.214
Genistein, ug/g, Mean (S.E)	141.77 (16.38)	164.75 (51.92)	137.93 (19.27)	0.655
Equol, ug/g, Mean (S.E)	68.03 (20.65)	88.37 (33.50)	64.63 (23.77)	0.579
O-desmethylangolensin, ug/g, Mean (S.E)	90.21 (10.03)	162.26 (48.08)	78.18 (10.23)	0.107
Enterodiol, ug/g, Mean (S.E)	111.83 (13.33)	117.46 (22.60)	110.89 (14.79)	0.800
Enterolactone, ug/g, Mean (S.E)	845.38 (84.86)	877.75 (119.89)	839.97 (92.97)	0.783
Total energy, kcal, Mean (S.E)	1920.02 (22.88)	1793.11 (55.60)	1941.21 (26.49)	0.024
Fiber, gm, Mean (S.E)	13.69 (0.22)	13.92 (0.76)	13.66 (0.24)	0.748

*GED* General Equivalent Diploma, *AA* Associate of Arts, *PIR* poverty-to-income ratio, *BMI* body mass index, *SE* standard error, *UL* uterine leiomyomata

Other race^a^ = Mexican American, other Hispanic and other race- Including Multi-Racial

Other^b^ = widowed, divorced, separated and living with partner

### Correlation between single metabolites of urinary phytoestrogens and UL

**Fig. 2:**
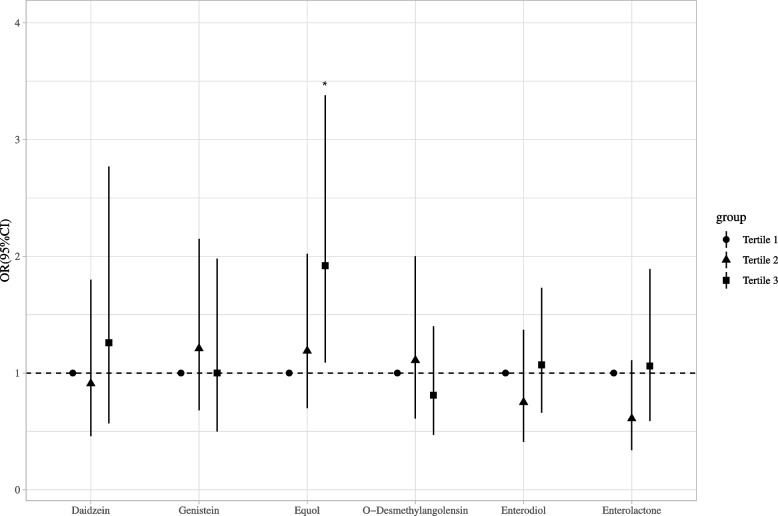
The association between single metabolite of urinary phytoestrogens and uterine leiomyomata in women in the multivariable logistic regression model. Other metabolites of urinary phytoestrogen were further adjusted for age, race/ethnicity, marital status, drinking status, body mass index, waist circumference, menopausal status, ovary removed status, use of female hormones, hormones/hormone modifiers and total energy

As shown in Supplemental Table 3, the result of univariate logistic regression indicated that age, race/ethnicity, marital status, drinking status, BMI, waist circumference, menopausal status, ovary removed status, use of female hormones, hormones/hormone modifiers and total energy might be covariates for this current study. The weighted logistic regression was used to assess the individual effect of each metabolite on UL (Table 2). After adjusting for age, race/ethnicity, marital status, drinking status, BMI, waist circumference, menopausal status, ovary removed status, use of female hormones, hormones/hormone modifiers and total energy, equol in the tertile 3 showed significant association with UL (Model 1: OR = 1.92, 95%CI: 1.07–3.43, *P* = 0.029). After further adjusting for age, race/ethnicity, marital status, drinking status, BMI, waist circumference, menopausal status, ovary removed status, use of female hormones, hormones/hormone modifiers, total energy, daidzein, genistein, O-desmethylangolensin, enterodiol, and enterolactone, the association of equol in the tertile 3 with UL remained significant (Model 2: OR = 1.92, 95%CI: 1.09–3.38,* P* = 0.024; Fig. 2).

**Table 2:** The individual effect of each metabolite on UL by using weighted logistic regression

Metabolites of urinary phytoestrogen	Model 1		Model 2	
	OR (95% CI)	*P*	OR (95% CI)	*P*
Daidzein				
Tertile 1	Ref		Ref	
Tertile 2	0.93 (0.52–1.68)	0.816	0.91 (0.46–1.80)	0.787
Tertile 3	1.13 (0.69–1.85)	0.625	1.26 (0.57–2.77)	0.565
Genistein				
Tertile 1	Ref		Ref	
Tertile 2	1.24 (0.77–1.98)	0.369	1.21 (0.68–2.15)	0.507
Tertile 3	1.15 (0.69–1.91)	0.594	1.00 (0.50–1.98)	0.989
Equol				
Tertile 1	Ref		Ref	
Tertile 2	1.17 (0.71–1.94)	0.533	1.19 (0.70–2.02)	0.506
Tertile 3	1.92 (1.07–3.43)	0.029	1.92 (1.09–3.38)	0.024
O-desmethylangolensin				
Tertile 1	Ref		Ref	
Tertile 2	1.21 (0.67–2.18)	0.528	1.11 (0.61–2.00)	0.729
Tertile 3	1.03 (0.63–1.69)	0.891	0.81 (0.47–1.40)	0.449
Enterodiol				
Tertile 1	Ref		Ref	
Tertile 2	0.78 (0.43–1.41)	0.407	0.75 (0.41–1.37)	0.347
Tertile 3	1.18 (0.74–1.90)	0.476	1.07 (0.66–1.73)	0.772
Enterolactone				
Tertile 1	Ref		Ref	
Tertile 2	0.65 (0.37–1.15)	0.139	0.61 (0.34–1.11)	0.107
Tertile 3	1.16 (0.68–2.00)	0.578	1.06 (0.59–1.89)	0.852

*UL* uterine leiomyomata, *Ref* reference, *OR* odds ratio, *CI* confidence interval

Model 1: adjusted age, race/ethnicity, marital status, drinking status, body mass index, waist circumference, menopausal status, ovary removed status, use of female hormones, hormones/hormone modifiers and total energy

Model 2: further adjusted for other metabolites of urinary phytoestrogen on the basis of Model 2

### WQS, qgcomp and BKMR models to assess the combined association between six metabolites and UL

**Fig. 3:**
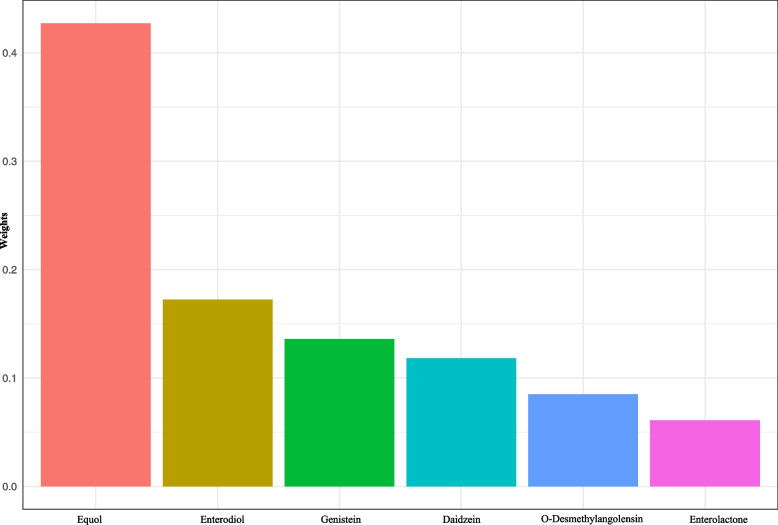
WQS model regression index weights for uterine leiomyomata. Model was adjusted for age, race/ethnicity, marital status, drinking status, body mass index, waist circumference, menopausal status, ovary removed status, use of female hormones, hormones/hormone modifiers and total energy

The WQS model was employed to estimate the combined effect of six metabolites of urinary phytoestrogen on UL. In the adjusted model (Table 3), mixed metabolites of urinary phytoestrogen had a positive association with UL (*P* = 0.011), and a tertile increase in the WQS index was related to a 68% increased risk of UL (95%CI: 1.12–2.51). We also calculated the estimated chemical weights of for each WQS index (Fig. 3). The highest weighted chemical in the WQS model was equol, followed by enterodiol and genistein.

**Table 3:** WQS model to estimate association between six metabolites and UL

Outcome	OR (95% CI)	*P*
Positive weight		
UL	1.68 (1.12–2.51)	0.011
Negative weight		
UL	1.14 (0.81–1.62)	0.448

*WQS* weighted quantile sum, *UL* uterine leiomyomata, *CI* confidence interval, *OR* odds ratio, OR estimates represent the odds ratios of UL when the WQS index was increased by one tertile; The positive and negative association was estimated respectively. Model was adjusted for age, race/ethnicity, marital status, drinking status, body mass index, waist circumference, menopausal status, ovary removed status, use of female hormones, hormones/hormone modifiers and total energy

**Fig. 4:**
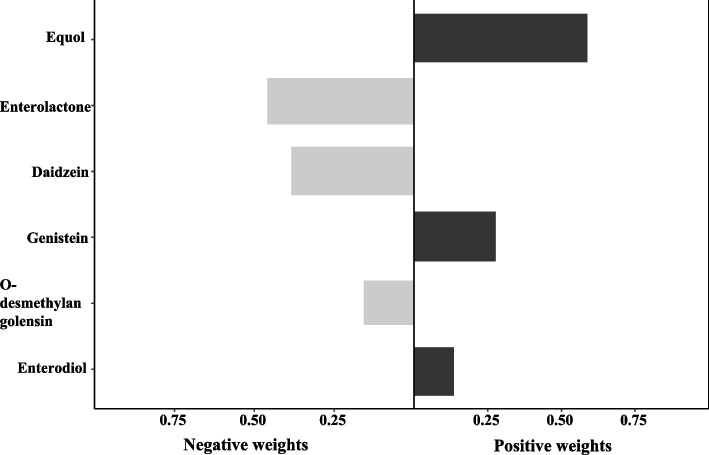
gqcomp model regression index weights of the mixture on uterine leiomyomata risk. Model was adjusted for age, race/ethnicity, marital status, drinking status, body mass index, waist circumference, menopausal status, ovary removed status, use of female hormones, hormones/hormone modifiers and total energy

Similar to the WQS model, a tertile increase in the gpcomp index was associated with risk of UL in the adjusted model (Table 4, OR = 1.51, 95%CI: 1.05–2.18, *P* = 0.027). Figure 4 shows the estimated weight of each metabolite on the UL risk. Equol had the largest positive weight, followed by genistein and enterodiol, respectively.

**Table 4:** Qgcomp model to assess the combined association between six metabolites and UL

Outcome	OR (95% CI)	*P*
g-computation index’s	1.51 (1.05–2.18)	0.027

*CI* confidence interval, *OR* odds ratio; Model was adjusted for age, race/ethnicity, marital status, drinking status, body mass index, waist circumference, menopausal status, ovary removed status, use of female hormones, hormones/hormone modifiers and total energy

**Fig. 5:**
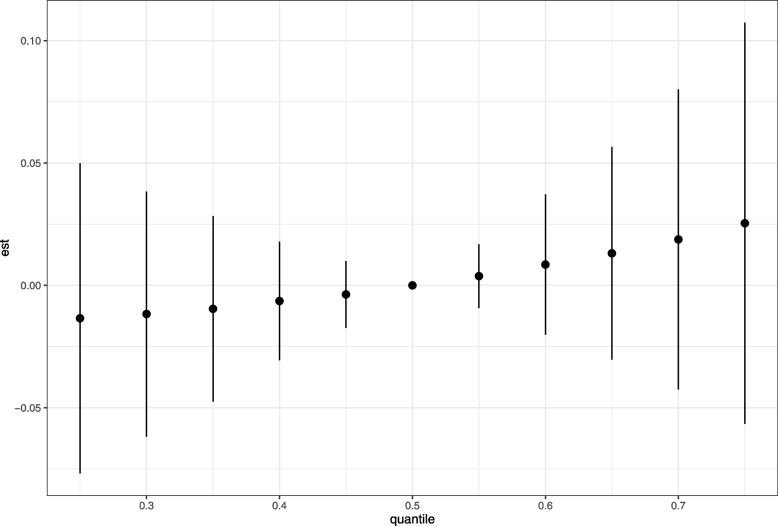
Combined effects of six metabolites of urinary phytoestrogens on uterine leiomyomata risk. Model was adjusted for age, race/ethnicity, marital status, drinking status, body mass index, waist circumference, menopausal status, ovary removed status, use of female hormones, hormones/hormone modifiers and total energy

Supplemental Table 4 summarizes the GroupPIP and CondPIP derived from the BKMR model for six metabolites. The GroupPIP of two group (genistein, equol and enterodiol; 0.34) was higher than one group (daidzein, O-desmethylangolensin, and enterolactone; 0.04). Enterodiol (CondPIP = 0.89) contributed most to the model for the UL risk. Figure 5 indicates the overall associations between six metabolites and UL risk. Although the high concentrations of all metabolites were not statistically different compared to their 50th percentile, the overall effect on UL of the mixture of exposures at the 60th and above quantiles showed an upward trend. As all other metabolites were at their median levels, equol and enterodiol have positive correlation on UL risk, while enterolactone has negative correlation (Supplemental Fig. 1). In addition, we also found that there may be an interaction between enterodiol and enterolactone on UL risk (Supplemental Fig. 2).

## Discussion

In this study including 1,579 US women, we assessed the relationship of urinary phytoestrogens and UL risk by using a number of statistical models. Overall, the weighted multivariate logistic regression indicated a correlation between equol and UL risk. By the WQS and gpcomp models, we observed a positive association between mixed metabolites of urinary phytoestrogen and UL risk. WQS model further identified that equol made the most contribution in the association between metabolite mixture of urinary phytoestrogen and UL risk. In the BKMR model, there was no significant association between overall mixed metabolites and UL appeared, but there was a trend towards an increase. Additionally, equol and enterodiol also showed a positive correlation with UL risk in gpcomp and BKMR models.

Previous studies have focused on the relationship between individual chemicals and health outcomes, but in fact, humans are often exposed to mixtures of multiple pollutants/chemicals [19, 22]. In recent years, several novel statistical methods have been developed to assess the impact of exposure to chemical mixtures on health outcomes, including WQS regression [17–19], gpcomp [20] and BKMR [21]. A review assessed the relationship between exposure to mixtures of per- and polyfluoroalkyl substances and adverse health outcomes, and highlighted the importance of WQS and BKMR for assessment of the effects of exposure to mixtures [23]. In addition, a cross-sectional study performed in US population found a positive association between combined exposures to mercury, arsenic, cadmium and lead measured in urine and higher estimated glomerular filtration rate using WQS regression [24], and they also indicated that there might be influence for exposure to multiple metals on kidney function. In the study of Zhang Y, et al., they reported that mixed exposure of ten commonly exposed endocrine-disrupting chemicals had a significant positive association with UL in WQS and BKMR models, the weight distribution showed the highest weights for mercury (weight = 0.35) and equol (weight = 0.29) [10]. However, to our knowledge, the association between the mixed metabolites of urinary phytoestrogen and UL has not been studied so far.

Unlike previous study [5, 10, 23], this study considered the mixed effect of six metabolites of urinary phytoestrogen (daidzein, genistein, equol, O-desmethylangolensin, enterodiol, and enterolactone) on UL risk by three approaches (WQS regression, qgcomp, and BKMR). These results also indicated that mixed metabolites of urinary phytoestrogen were positively linked to the UL risk, with the greatest effect being from equol. Equol was related to an increased risk of UL. Our results are also consistent with previous study [10]. Equol, a metabolite of soy isoflavone daidzein, has estrogenic and antioxidant activity [25]. Several studies have showed that equol has a beneficial impact on metabolic diseases [26, 27]. But, estrogen-dependent diseases such as UL, are likely to be exacerbated by the estrogenic effects of equol. As described in an animal study, equol may trigger uterine tissue hyperplasia by increasing luminal epithelial cell height and myometrial and stromal thickness, which further lead to UL [28]. Our results agree with a previous study that estradiol could stimulate growth of UL, and was considered to be associated with increased risk of UL [29]. Although we found a combined effects of mixed metabolites on UL risk, the molecular mechanism related to the relationship of phytoestrogen and UL remains unclear. Further exploration is needed regarding the potential mechanisms in the association.

The main strength of this study was the use of WQS regression, qgcomp, and BKMR, which allowed us to assess the mixed metabolites of urinary phytoestrogen and UL risk. Some limitations for this study should be considered. First of all, because of the design of this cross-sectional study, there was a limitation in the causal relationship between urinary phytoestrogens and UL. Second, some possible confounders were lacking in this NHANES database, such as family history of UL. We did not adjust for history of hysterectomy because they may be a consequence of the outcome [30]. Third, for participants in the NHANES database, a single spot urine sample was only collected for metabolites analysis. The concentrations of metabolites of phytoestrogens may vary over time. Fourth, we excluded 4,587 women who were not measurement of urinary phytoestrogen concentrations. Urinary phytoestrogens were tested in 1/3 of the participants aged 6 years and older in the NHANES database. However, this study considered the weights in the analysis, so the bias was relatively small. Prospective studies with large sample size are warranted to further analyze the relationship of urinary phytoestrogens and UL, and the related mechanisms.

## Conclusion

In summary, our results implied an association of equol and UL. Importantly, WQS regression, qgcomp, and BKMR models was adopted to analyze the combined effects of mixed metabolites on UL risk. A positive association between the mixed metabolites of urinary phytoestrogen and UL was also identified, with the greatest contribution from equol. This study provides evidence that urinary phytoestrogen-metabolite mixture was closely related to the risk of female UL and further research is needed to explore the detailed mechanism.

## Supplementary Information

**Additional file 1: Supplemental Table 1.** Distribution of urinary phytoestrogen levels. **Supplemental Table 2.** Sensitivity analysis of data before and after interpolation. **Supplemental Table 3.** The Selection of covariates by univariate logistic regression. **Supplemental Table 4.** GroupPIP and CondPIP of six metabolites.

**Additional file 2: Supplemental Fig. 1.** Univariate exposure–response functionbetween metabolite exposure and UL with fixing all the other metabolites at their median level. Model was adjusted for age, race/ethnicity, marital status, drinking status, body mass index, waist circumference, menopausal status, ovary removed status, use of female hormones, hormones/hormone modifiers and total energy.

**Additional file 3: Supplemental Fig. 2.** Bivariate exposure–response function for metabolites in UL, with exposure 1 metabolite at its 10%, 50%, and 90% levels and other metabolites fixed at their median levels. Model was adjusted for age, race/ethnicity, marital status, drinking status, body mass index, waist circumference, menopausal status, ovary removed status, use of female hormones, hormones/hormone modifiers and total energy.

## Data Availability

The datasets generated and/or analyzed during the current study are available in the NHANES database, https://wwwn.cdc.gov/nchs/nhanes/.

## Abbreviations

UL: Uterine leiomyomata
NHANES: National Health and Nutrition Examination Survey
NCHS: National Center for Health Statistics
HPLC: High-performance liquid chromatography
MS: Mass spectrometric
BMI: Body mass index
SE: Standard error
OR: Odds ratio
CI: Confidence interval
WQS: Weighted quantile sum
