# Supplementary material for: Urinary phytoestrogens and the risk of uterine leiomyomata in US women
Source: BMC Womens Health. 2023 May 13;23:261. doi: 10.1186/s12905-023-02381-5 (PMC10182647; doi:10.1186/s12905-023-02381-5)
Supplement: Supplementary file 1 — Additional file 1: Supplemental Table 1. Distribution of urinary phytoestrogen levels. Supplemental Table 2. Sensitivity analysis of data before and after interpolation. Supplemental Table 3. The Selection of covariates by univariate logistic regression. Supplemental Table 4. GroupPIP and CondPIP of six metabolites. [file 12905_2023_2381_MOESM1_ESM.docx]

**Supplemental Table 1 Distribution of urinary phytoestrogen levels (ug/g creatinine)**

| **Metabolites of urinary phytoestrogen** | **Geometric mean (95% CI)** | **Tertile 1** | **Tertile 2** | **Tertile 3** |
| --- | --- | --- | --- | --- |
| Daidzein | 57.78 (51.51-64.07) | $\leq$24.92 | 24.92 ~ 109.00 | >109.00 |
| Genistein | 27.61 (24.36-30.85) | ≤12.32 | 12.32 ~ 49.39 | >49.39 |
| Equol | 8.60 (7.71-9.48) | ≤5.05 | 5.05 ~ 12.98 | >12.98 |
| O-desmethylangolensin | 4.78 (4.13-5.43) | ≤1.27 | 1.27 ~ 11.53 | >11.53 |
| Enterodiol | 37.69 (33.82-41.56) | ≤25.00 | 25.00 ~ 75.44 | >75.44 |
| Enterolactone | 270.19 (233.93-306.44) | ≤199.31 | 199.31 ~ 627.78 | >627.78 |

**Supplemental Table 2 Sensitivity analysis of data before and after interpolation**

| **Variables** | **Missing proportion** | **Before interpolation** | **After interpolation** | ***P*** |
| --- | --- | --- | --- | --- |
| Education level, n (%) | 0.06% |  |  | 0.402 |
| Less Than 9th Grade |  | 121 (3.42) | 121 (3.42) |  |
| 9-11th Grade |  | 241 (10.37) | 241 (10.36) |  |
| High School Grad/GED or Equivalent |  | 334 (22.32) | 334 (22.32) |  |
| Some College or AA degree |  | 516 (37.07) | 516 (37.07) |  |
| College Graduate or above |  | 366 (26.82) | 367 (26.83) |  |
| PIR, Mean (S.E) | 5.26 % | 3.06 (0.07) | 3.05 (0.07) | 0.623 |
| Waist circumference, cm, Mean (S.E) | 0.82% | 92.56 (0.59) | 92.67 (0.59) | 0.068 |
| Total energy, kcal, Mean (S.E) | 1.65% | 1918.18 (23.17) | 1920.02 (22.88) | 0.376 |
| Fiber, gm, Mean (S.E) | 1.65% | 13.68 (0.22) | 13.69 (0.22) | 0.579 |

GED=General Equivalent Diploma, AA= Associate of Arts, PIR=poverty-to-income ratio, SE=standard error.

**Supplemental Table 3 The Selection of covariates by univariate logistic regression**

| Variables | OR (95% CI) | *P* |
| --- | --- | --- |
| Age | 1.10 (1.07-1.12) | <0.001 |
| Race/ethnicity |  |  |
| Non-Hispanic White | Ref |  |
| Non-Hispanic Black | 1.98 (1.39-2.82) | <0.001 |
| Other race^#^ | 0.67 (0.41-1.11) | 0.110 |
| Marital status |  |  |
| Married | Ref |  |
| Never married | 0.46 (0.25-0.85) | 0.011 |
| Other^*^ | 0.94 (0.62-1.43) | 0.760 |
| Education level |  |  |
| High school and below | Ref |  |
| High school Grad/ GED or Equivalent | 1.21 (0.66-2.20) | 0.528 |
| Some College or AA degree/College Graduate or above | 1.22 (0.68-2.19) | 0.503 |
| PIR |  |  |
| <1.0 | Ref |  |
| ≥1.0 | 1.52 (0.89-2.59) | 0.115 |
| Smoking status |  |  |
| No | Ref |  |
| Yes | 1.35 (0.91-2.02) | 0.132 |
| Drinking status |  |  |
| No | Ref |  |
| Yes | 1.44 (0.99-2.08) | 0.049 |
| BMI | 1.03 (1.01-1.05) | 0.003 |
| Waist circumference | 1.01 (1.01-1.02) | 0.012 |
| Cotinine | 1.01 (1.01-1.01) | 0.804 |
| Age at menarche | 0.94 (0.85-1.04) | 0.235 |
| Menopausal status |  |  |
| No | Ref |  |
| Yes | 3.56 (2.22-5.72) | <0.001 |
| Ovary removed status |  |  |
| No | Ref |  |
| Yes | 12.23 (7.50-19.93) | <0.001 |
| Use of female hormones |  |  |
| No | Ref |  |
| Yes | 2.23 (1.59-3.21) | <0.001 |
| Use of other hormonal drugs |  |  |
| No | Ref |  |
| Yes | 1.79 (1.02-3.14) | 0.039 |
| Use of non-steroidal anti-inflammatory drugs |  |  |
| No | Ref |  |
| Yes | 1.08 (0.49-2.36) | 0.844 |
| Pregnancy status |  |  |
| No | Ref |  |
| Yes | 0.43 (0.16-1.18) | 0.094 |
| Number of gravidities |  |  |
| 1 | Ref |  |
| >1 | 1.64 (0.96-2.80) | 0.065 |
| Unknown | 0.69 (0.31-1.54) | 0.358 |
| Total energy | 0.99 (0.99-0.99) | 0.032 |
| Fiber | 1.01 (0.98-1.03) | 0.744 |

Other race ^#^=Mexican American, other Hispanic and other race- Including Multi-Racial;

Other* =widowed, divorced, separated and living with partner;

GED=General Equivalent Diploma, AA= Associate of Arts, PIR=poverty-to-income ratio, BMI= body mass index, OR=odds ratio, CI=confidence interval, Ref=reference.

**Supplemental Table 4** **GroupPIP and CondPIP of six metabolites**

| Variables | group | GroupPIP | CondPIP |
| --- | --- | --- | --- |
| Daidzein | 1 | 0.04 | 0.10 |
| Genistein | 2 | 0.34 | 0.01 |
| Equol | 2 | 0.34 | 0.11 |
| O-Desmethylangolensin | 1 | 0.04 | 0.01 |
| Enterodiol | 2 | 0.34 | 0.89 |
| Enterolactone | 1 | 0.04 | 0.89 |

GroupPIP =Group posterior inclusion probability; CondPIP =Conditional posterior inclusion probability.
