# Supplementary figures and images for: Urinary phytoestrogens and the risk of uterine leiomyomata in US women
Source: BMC Womens Health. 2023 May 13;23:261. doi: 10.1186/s12905-023-02381-5 (PMC10182647; doi:10.1186/s12905-023-02381-5)

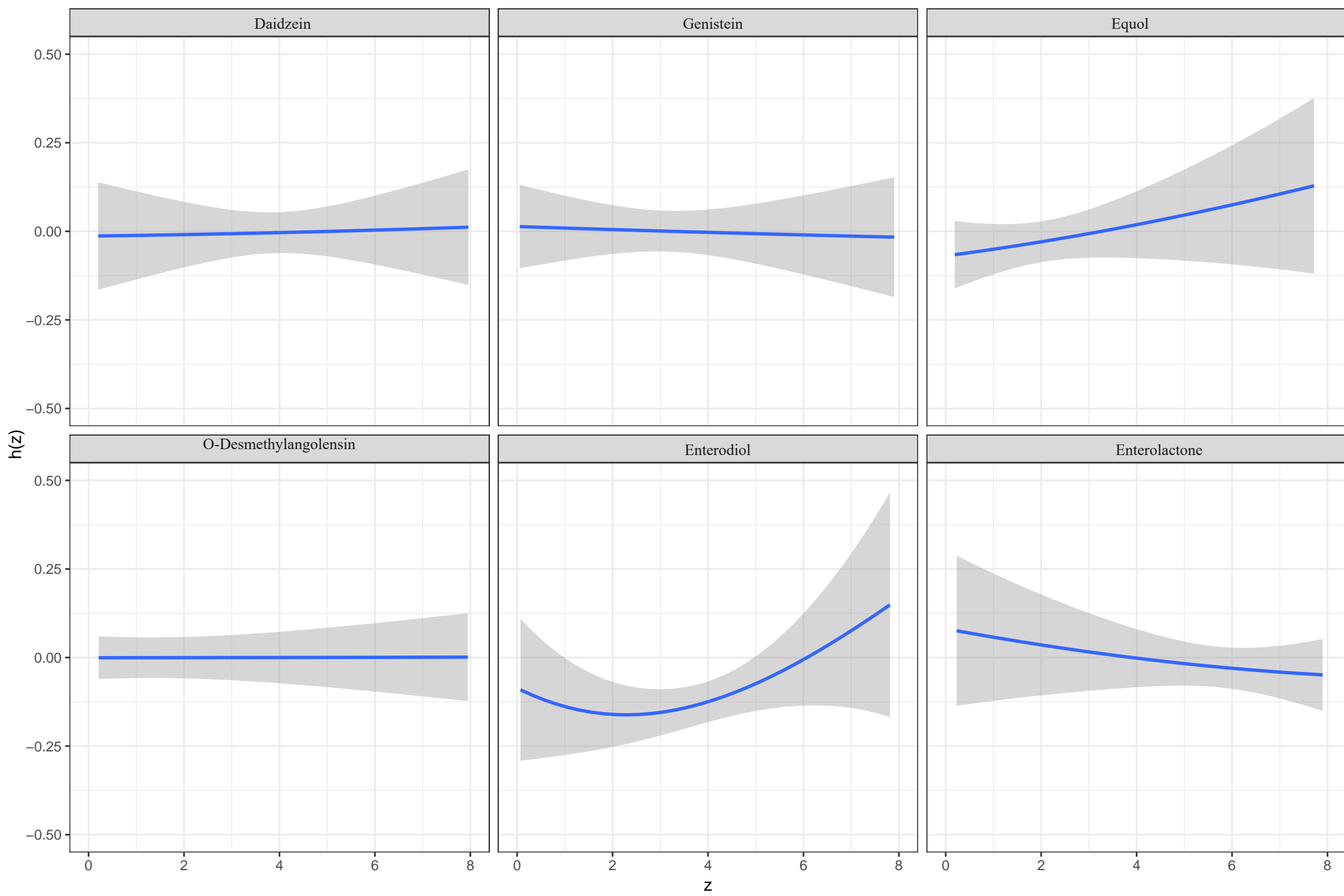

Supplement: Supplementary file 2 — Additional file 2: Supplemental Fig. 1. Univariate exposure–response functionbetween metabolite exposure and UL with fixing all the other metabolites at their median level. Model was adjusted for age, race/ethnicity, marital status, drinking status, body mass index, waist circumference, menopausal status, ovary removed status, use of female hormones, hormones/hormone modifiers and total energy. [file 12905_2023_2381_MOESM2_ESM.pdf]

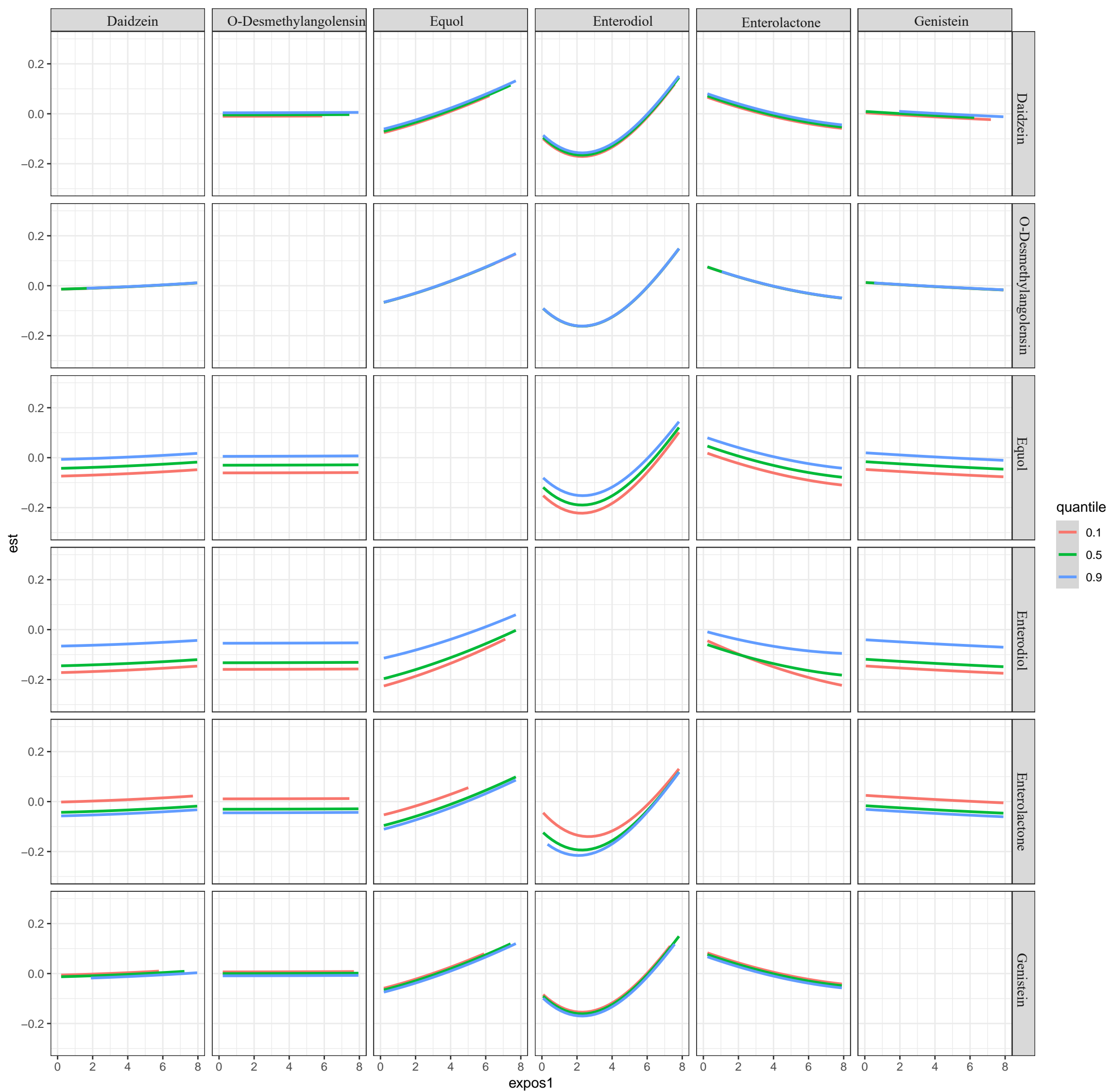

Supplement: Supplementary file 3 — Additional file 3: Supplemental Fig. 2. Bivariate exposure–response function for metabolites in UL, with exposure 1 metabolite at its 10%, 50%, and 90% levels and other metabolites fixed at their median levels. Model was adjusted for age, race/ethnicity, marital status, drinking status, body mass index, waist circumference, menopausal status, ovary removed status, use of female hormones, hormones/hormone modifiers and total energy. [file 12905_2023_2381_MOESM3_ESM.pdf]
